# Complete mitochondrial genomes of two damselfly species in coenagrionidae and phylogenetic implications

**DOI:** 10.1080/23802359.2021.1955635

**Published:** 2021-07-25

**Authors:** Bin Jiang, Jia Li, Yongmei Zhang, Yang Sun, Shulin He, Guozhi Yu, Guosheng Lv, Dirk J. Mikolajewski

**Affiliations:** a College of Life Science, Anhui Normal University, Wuhu, China; b Faculty of Forestry and Wood Sciences, Czech University of Life Science Prague, Prague, Czech Republich; c College of Life Science, Sichuan Agricultural University, Ya’an, China; d Institut für Biologie, Freie Universität Berlin, Berlin, Germany

**Keywords:** Coenagrionidae, damselfly, mitogenomes, phylogeny

## Abstract

*Agriocnemis femina* (Brauer, 1868) and *Ischnura senegalensis* (Rambur, 1842) are two damselflies inhabiting paddy lands. As an intermediate predator, they play an important role in controlling certain crop pest and mosquitoes. In this study, we sequenced complete mitogenomes of these two species. The total length of mitogenomes is 15,936 bp in *A. femina* and 15,762 bp in *I. senegalensis*. Both of mitogenomes consist of 13 protein-coding genes, 22 tRNA genes, two rRNA genes, and one control region. The close relationship between *I. senegalensis* and *I. elegans* was further proved by phylogenetic analysis. Our phylogenetic analysis indicated a clear two lineages in Coenagrionidae (Core and ridge-faced Coenagrionidae). Ridge-faced Coenagrionidae consisted of *Megaloprepus caerulatus* and *Ceriagrion fallax*. In core Coenagrionidae, *Ischnura* and *Enallagma* are most closely related; they formed one clade with *Agriocnemis* and then grouped together with *Paracerion*. Our study provides new genetic information for further study in phylogenetic analysis of Coenagrionidae.

Odonates represent a prime group of non-model organisms that are widely used to study and answer key questions in ecology and evolution (Zaka et al. 2014). For instance, odonates are amongst the most ancient groups of insects and own a crucial position in the evolution of winged insects (Bechly 1995). In suborder Zygoptera, Coenagrionidae includes more than 1100 species, which is a diverse damselfly family with worldwide distribution. In this study, we sequenced and characterized two mitogenomes from the damselfly family Coenagrionidae (*Agriocnemis femina,* Brauer, 1868 and *Ischnura senegalensis,* Rambur, 1842). Both of these two species inhabit paddy land and become important predators for biological control of crop pests or mosquitoes (Kandibane et al. 2005).

The adult damselflies of *A. femina* (Brauer, 1868) and *I. senegalensis* (Rambur, 1842) were collected by sweep net in the Shenshan park of Wuhu, China (118°24’53” E, 31°20’51” N for *A. femina* and 118°24’57” E, 31°20’58” N for *I. senegalensis*) in July, 2019 and preserved in 90%-ethanol at room temperature. The specimen was deposited at Entomological Evolution and Ecology Room in Anhui Normal University (Bin Jiang, bin.jiang@ahnu.edu.cn) under the voucher number L502-SS03. Genomics DNA was extracted from muscle tissues of thorax using CTAB methods (Bechly 1995). The quality of DNA was checked by using NanoDrop and measured by Qubit. Samples were sent for library preparation and paired-end sequencing with Novaseq following the standard protocol from Illumina in BenaGen Inc. (Wuhan, China). The adapters and low-quality reads in raw data were trimmed and filtered by using SOAPnuke 1.3.0. Genome assemblies were conducted using SPAdes 3.13.0 (parameter: -k 127) (Dierckxsens et al. 2016). We used MITOS web server (http://mitos.bioinf.uni-leipzig.de) to annotate genomes (Bernt et al. 2013).

The total length of the new mitogenomes in our study is 15,936 bp in *A. femina* (Accession number in Genbank: MT787566) and 15,762 bp in *I. senegalensis* (Accession number in Genbank: MT787567). We noticed that a partial mitogenome of *A. femina* (13,280 bp, accession number in Genbank: MK951667) is reported earlier. Comparing MK951677, 20 SNPs were found; one T-deletion in the interspace between *trnT* and *trnP* and one A-deletion and two A-insertions in 16S RNA coding region in MT787566 were found. Like other mitogenomes in Coenagrionidae, both new mitogenomes from us contain 13 protein-coding genes, 22 tRNA genes, two rRNA genes, and one control region. All tRNA sequences length range from 63 to 72 bp. In both mitogenomes, the shortest tRNA sequence is *trnG* and the longest one is *trnK*. According to the predicted tRNA structure from MITOs, all tRNA sequences can form the characteristic clover leaf secondary structures. In both mitogenomes, *trnS1* lacks DHU-stem and *trnF* lacks TΨC loop. For protein-coding genes, in *A. femina* eight genes (*cox1, cox2, atp6, cox3, nad3, nad4, nad4L,* and *cob*) use ATG/ATA (encoding for methionine) and 5 genes (*nad2, atp8, nad5, nad6* and *nad1*) use ATT (encoding for isoleucine) as start codon; in *I. senegalensis*, *nad6* use ATA and *nad3* use ATT as start codon and the other genes are the same as in *A. femina*. We identified a standard stop codon for most of protein-coding genes; however, *cox3* and *nad5* in *A. femina* and *nad5* in *I. senegalensis* had an incomplete stop codon.

In order to determine the phylogenetic relationship of *A. femina* and *I. senegalensis* in Coenagrionidae, we collected 22 other mitogenome sequences from GenBank in Zygoptera (Lin et al. 2010; Lorenzo-Carballa et al. 2014; Chen et al. 2015; Feindt et al. 2016; Okuyama and Takahashi 2017; Zhang et al. 2017; Xu et al. 2018; Lan et al. 2019; Song et al. 2019; Wang et al. 2019; Shao et al. 2021) and two outgroup species from Anisoptera (*Anax imperator* (Herzog et al. 2016); *Brachythemis contaminata* (Yu et al. 2016)). We reconstructed the Maximum-likelihood tree by iqtree 2.1.2 (Nguyen et al. 2015) (Figure 1). To assess ML nodal support, we calculated 1000 likelihood bootstrap replications. Bayesian analyses were conducted with MrBayes v3.2 (Ronquist et al. 2012). The GTR + I+G substitution model was identified as the best model by Modeltest 3.7 (Posada & Crandall 1998). MCMC were performed for 100,000 generations and terminated after the average split frequencies falling below 0.01. Trees were sampled every 100 replicates with the first 25% of samples discarded as burn-in. Both phylogeny analyses indicated that Coenagrionidae separated into core Coenagrionidae and ridge-faced Coenagrionidae two lineages (Dijkstra et al. 2014). Newly sequenced *I. senegalensis* has a close phylogenetic relationship with *I. elegans* and forms one clade with *I. pumilio*. The close relationship between *I. elegans* and *I. senegalensis* can also be proved by their interspecific hybridization in the lab (Okude et al. 2020). *Ischnura* species formed one clade with *Enallagma* species and then clustered with newly sequenced *A. femina*. Therefore, the complete mitogenomes of *I. senegalensis* and *A. femina* in Coenagrionidae provide valuable genetic information for further phylogenetic analyses in Zygoptera.

**Figure 1.**
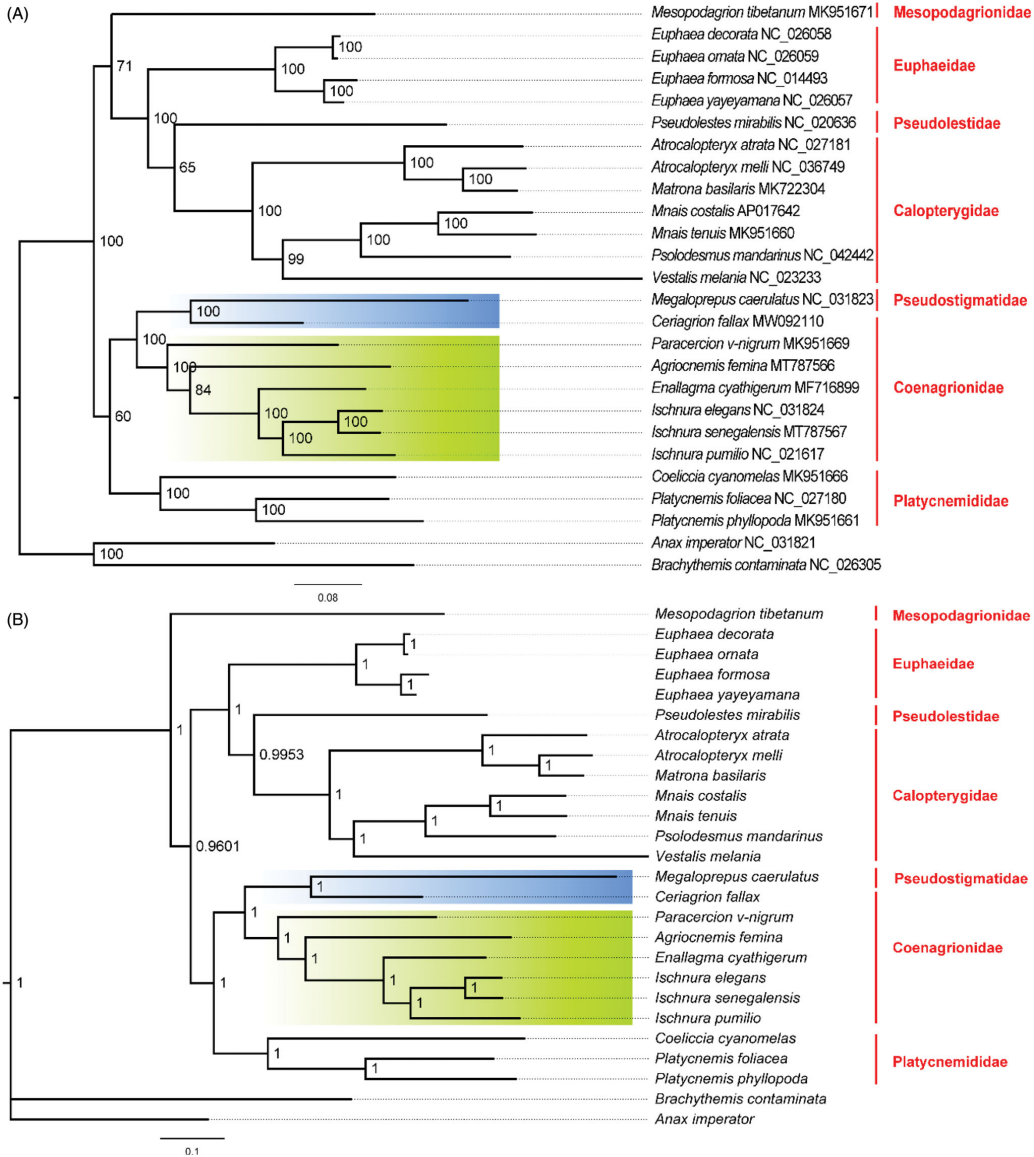
Phylogenetic relationships based on 13 mitochondrial protein-coding genes in Zygoptera. Branches with green background indicate core Coenagrionidae and branches with blues background represent ridge-faced Coenagrionidae. Nodal values indicate (A) bootstrap support values in ML tree and (B) the posterior probabilities in Bayes tree.

## Data Availability

The data that support the findings of this study are openly available in NCBI (National Center for Biotechnology Information) at https://www.ncbi.nlm.nih.gov/, reference number MT787566 and MT787567. The associated BioProject, SRA, and Bio-Sample numbers of *Ischnura senegalensis* are PRJNA730433, SRR14574107, and SAMN19231599. For *Agriocnemis femina*, submission numbers are successively PRJNA730438, SRR14574586 and SAMN19231647.
